# Masked diabetes insipidus in pituitary metastasis from breast cancer after thalamic biopsy: a case report

**DOI:** 10.1186/s13256-021-03229-y

**Published:** 2022-01-14

**Authors:** Hiroaki Hashimoto, Tomoyuki Maruo, Masami Nakamura, Yukitaka Ushio, Masayuki Hirata, Haruhiko Kishima

**Affiliations:** 1 Department of Neurosurgery, Otemae Hospital, Osaka, Osaka 540-0008 Japan; 2 Department of Neurological Diagnosis and Restoration, Graduate School of Medicine, Osaka University, Suita, Osaka 565-0871 Japan; 3 Department of Neurosurgery, Graduate School of Medicine, Osaka University, Suita, Osaka 565-0871 Japan

**Keywords:** Pituitary metastasis, Masked diabetes insipidus, Panhypopituitarism, Stereotactic biopsy, Breast cancer, Case report

## Abstract

**Background:**

Symptomatic pituitary metastasis is rare; furthermore, it can result in diabetes insipidus and panhypopituitarism. Since diabetes insipidus is masked by concurrent panhypopituitarism, it can impede the diagnosis of pituitary dysfunction.

**Case presentation:**

A 68-year-old Japanese female suffering from pituitary and thalamic metastases caused by untreated breast cancer, underwent a biopsy targeting the thalamus, not the pituitary. She lacked prebiopsy pituitary dysfunction symptoms; however, these symptoms unexpectedly occurred after biopsy. Diabetes insipidus was masked by corticosteroid insufficiency, and she showed normal urinary output and plasma sodium levels. Upon commencement of glucocorticoid replacement therapy, the symptoms of diabetes insipidus appeared.

**Conclusions:**

In this case, thalamic biopsy, as opposed to pituitary biopsy, was performed to preserve pituitary function. However, pituitary dysfunction could not be avoided. Caution is necessary for asymptomatic patients with pituitary metastases as invasive interventions, such as surgery, may induce pituitary dysfunction. Moreover, with respect to masked diabetes insipidus, there is a need to carefully consider pituitary dysfunction to avoid misdiagnosis and delayed treatment.

## Background

Pituitary metastases are rare, and are frequently asymptomatic or present with nonspecific symptoms [1]. Patients with symptomatic pituitary metastasis often have concurrent diabetes insipidus (DI) and panhypopituitarism, which are caused by adrenocorticotropic hormone (ADH) deficiency and pituitary anterior lobe dysfunction, respectively [2, 3]. As DI is masked by the concomitant deficiency of adrenocorticotropic hormone (ACTH), one of the hormones of the pituitary anterior lobe [3], there is a need to carefully consider “masked DI” to avoid misdiagnosis and delayed treatment.

This is a case of pituitary metastasis accompanied by thalamus metastasis, with breast cancer as the source. The patient underwent a biopsy targeting the thalamus, with no prebiopsy DI symptoms. We opted not to biopsy the pituitary metastases to avoid pituitary dysfunction. After the biopsy, there was transient polyuria, which improved within a few days. The patient presented with continuous lethargy and fatigue. We inferred that it was induced by the progression of metastasis and peritumoral edema, and glucocorticoids were administrated accordingly. However, DI symptoms appeared upon glucocorticoid replacement therapy. This suggested that thalamic biopsy induced pituitary dysfunction, resulting in DI and panhypopituitarism. We concluded that the continuous lethargy and fatigue were signs of panhypopituitarism as opposed to brain edema. Moreover, DI was masked by ACTH deficiency, which led to normal urinary output and plasma sodium levels. We report an illustrative case of pituitary metastasis showing masked DI.

## Case presentation

**Fig. 1:**
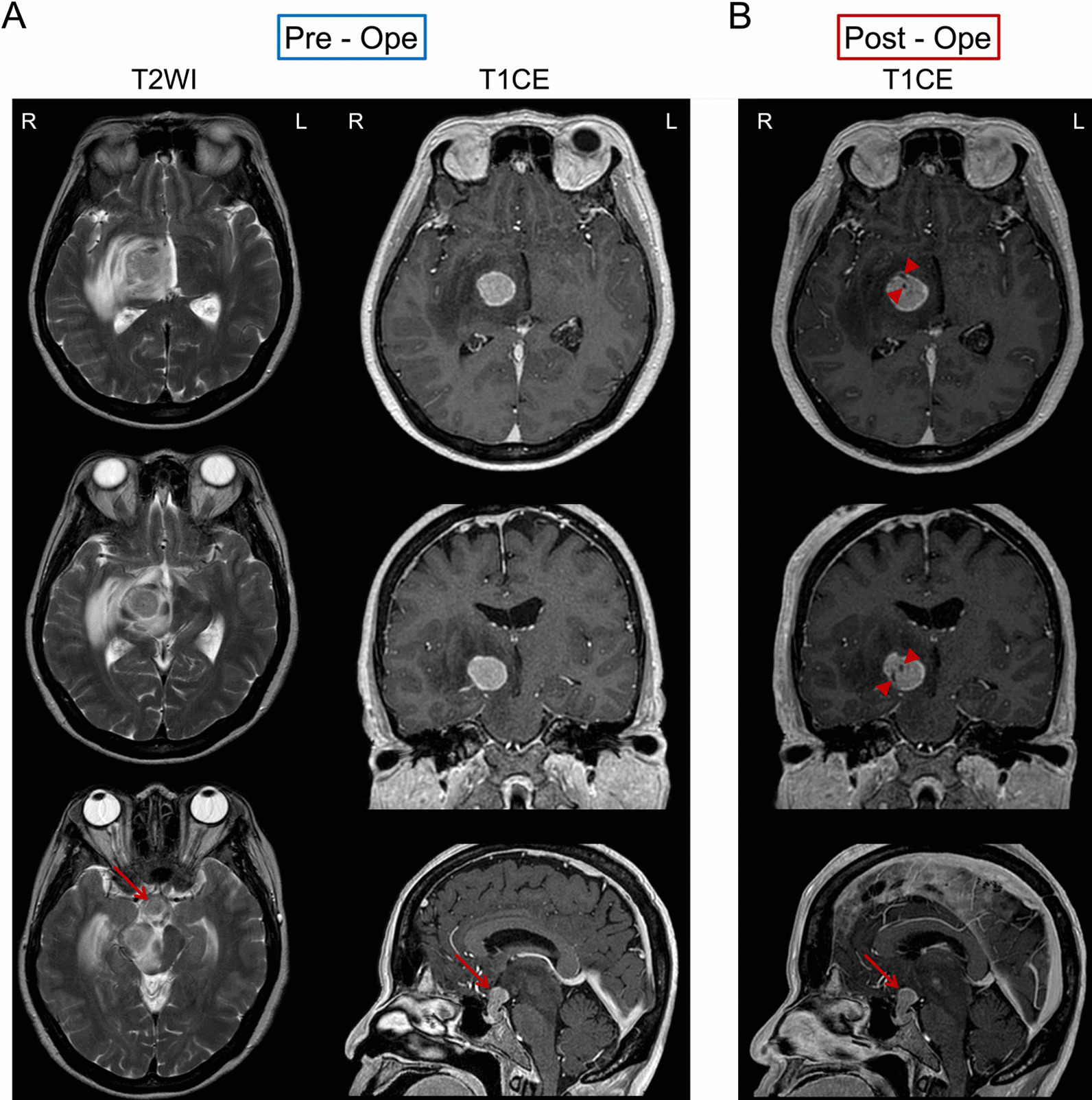
Pre- and postoperative MRI images. **A** High-density areas on T2WI (left column) are observed around the right thalamus lesion, with invasion of the right pedunculus cerebri. Lesions in the right thalamus and hypothalamic–pituitary axis have been shown on contrast-enhanced T1WI (right column). There is thickening of the pituitary stalk. **B** Postbiopsy contrast-enhanced T1WI targeting the right thalamus demonstrates the correct biopsy (scar due to biopsy indicated by red wedge arrows). Hypothalamic–pituitary axis lesions are indicated by red arrows

A 68-year-old Japanese woman presented to our hospital with a skilled movement disorder of the left hand, which had gradually progressed over the previous few months. She showed normal consciousness and slight fatigue without thirst. She had no psychological problems and no challenges regarding financial, language, and cultural matters. She previously underwent a uterine myoma surgery and had two sisters who had been diagnosed with breast cancer. Brain magnetic resonance imaging (MRI) revealed two mass lesions. One lesion was 21 × 21 × 17 mm and was located in the right thalamus with accompanying brain edema. On T2-weighted imaging (T2WI), this was shown as high-density areas that progressed to the right pedunculus cerebri. The other lesion was located in the hypothalamus, infundibular stalk, and pituitary posterior lobe, which compose the hypothalamic–pituitary axis (HPA). The stalk was thickened on T1-weighted imaging (T1WI), showing an absence of the normal posterior pituitary bright spot (PPBS). Contrast-enhanced T1WI showed intense enhancement along the lesion edges and uniform enhancement within the lesions (Fig. 1A). Furthermore, she had an undiagnosed and untreated left breast mass, which was considered as a breast cancer with accompanying axillary lymph node metastasis.

Upon hospital admission, her plasma sodium levels (140 mEq/l) were within the normal range. She underwent a stereotactic biopsy targeting the thalamus, but not HPA, lesions (Fig. 1B). Pathological examination revealed a carcinoma. Therefore, we considered that the pituitary and thalamus metastases were from the breast cancer which had not been previously treated. We opted to treat the metastasis before breast cancer treatment since the metastasis compressed the pyramidal tract.

**Fig. 2:**
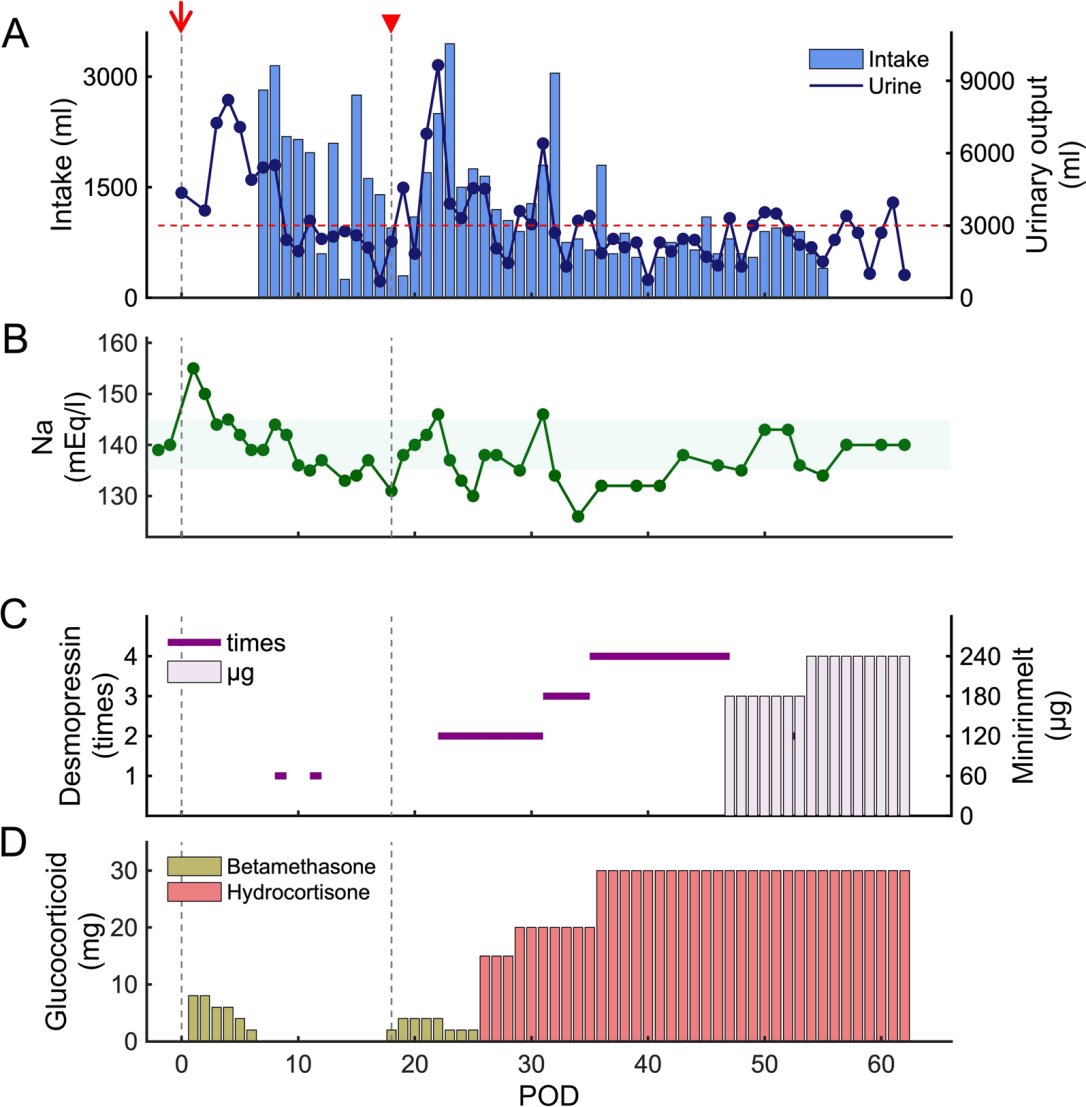
Clinical course. Daily urinary output (ml) and fluid intake (ml) (**A**); plasma sodium levels (mEq/l) (**B**); intranasal (times/day) and oral (μg) desmopressin administration (**C**); and glucocorticoid replacement (mg) (**D**). Operative days are indicated by a red arrow. Immediately after biopsy, urinary output was > 3000 ml (red dashed line in **A**) and there was a temporary increase in plasma sodium levels. We temporally administrated glucocorticoid for brain edema treatment (postoperative day 1–6). From postoperative day 9, urinary output was < 3000 ml and plasma sodium levels normalized. Due to possible worsening of brain edema, we administrated glucocorticoid at postoperative day 18 (red wedge arrow). Subsequently, there was a notable increase in daily urinary output. We could determine that central diabetes insipidus was masked by insufficient adrenocorticotropic hormone. Polyuria and plasma sodium levels were improved by desmopressin and glucocorticoid replacement. We instructed the patient to drink according to thirst; therefore, fluid intake volumes subsequently increased following an increased urinary output

After undergoing biopsy surgery, she complained of polyuria and thirst. At postoperative day (POD) 0, her urine volume was > 3000 ml. At POD 1, plasma sodium levels increased to 155 mEq/l (Fig. 2A, B). We suspected that the transient DI and polyuria were induced by biopsy-related stress and would improve within some days. As such, the patient was instructed to intake fluid as dictated by her thirst (Fig. 2A) and was prescribed desmopressin nasal spray for rescue use (POD 8 and 11 in Fig. 2C). Subsequently, her plasma sodium levels normalized, and her daily urine output improved to < 3000 ml/day. However, the left hemiplegia progressed and she had become bedbound. She also complained of fatigue and lethargy. We assumed that the metastasis had enlarged, with worsening brain edema. Consequently, glucocorticoid (betamethasone) was administrated (red wedge arrow in Fig. 2). After glucocorticoid administration, there was notable polyuria and elevation of plasma sodium levels. These symptoms suggested that central DI due to a metastatic pituitary tumor was masked by adrenal insufficiency, which was revealed by glucocorticoid replacement. In retrospect, the gradual decrease in plasma sodium levels was due to ACTH deficiency (POD 1–18 in Fig. 2B).

Regular desmopressin administration ameliorated the polyuria (Fig. 2A, C). Intranasal desmopressin was initially used due to easy regulation of the administration amount. Subsequently, oral formulations were used. We also prescribed tapered administration of betamethasone, followed by hydrocortisone administration for replacement therapy (Fig 2D) and an increase in plasma sodium levels (Fig. 2B). Conventional multiple fractionated radiation therapy was implemented from POD 27 to POD 47 (37.5 Gy/15 fr). Levothyroxine sodium hydrate was additionally administrated due to hypothyroidism from POD 50. Needle biopsy of the breast masses revealed carcinoma (T4N3M1, stage IV). The breast cancer had no surgery indication. Thus, abemaciclib and anastrozole were administered in addition to radiation therapy for the breast cancer (50 Gy/25 fr). The DI and panhypopituitarism were managed through the prescribed medications, and her fatigue and lethargy improved. However, she showed permanent left hemiplegia and died 9 months after the first admission due to the progression of the brain metastases.

## Discussion

This is a case of thalamic and pituitary metastasis from breast cancer with accompanying central DI and panhypopituitarism, from which two learning points can be derived. First, an invasive intervention, specifically stereotactic biopsy in this study, may induce pituitary dysfunction. Second, central DI is masked by comorbid ACTH insufficiency.

Numerous reports have indicated that metastasis to the pituitary gland is rare [1, 2, 4, 5]. Specifically, pituitary metastases accounted for 0.4–0.87% of intracranial metastases [6, 7]. While pituitary adenomas accounted for 84.6% of pituitary tumors, pituitary metastases occurred in 0.6% of cases [8]. Literature reviews for pituitary metastases showed that primary symptoms are visual involvement (49–62%), panhypopituitarism (38%), and DI (28–38%) [1, 9]. However, the frequent absence of symptoms makes diagnosis difficult, which delays detection of early-stage disease [1]. The primary malignant disease usually manifests first, followed by incidental detection of pituitary metastasis on imaging [10]. In this study, the primary malignancy was the breast cancer, reportedly one of the most common primary cancers for pituitary metastases [1, 7].

In this case, thalamic metastasis, as opposed to pituitary metastasis or the primary malignancy, caused the primary manifestation and chief complaint (that is, skilled movement disorder). The pituitary metastasis was asymptomatic before the biopsy surgery. As postoperative DI has been observed in 10.2% of endoscopic transsphenoidal surgery [11], the thalamic metastasis, as opposed to the pituitary metastasis, was selected as a biopsy target to preserve pituitary function. Nonetheless, postoperative pituitary dysfunction was observed, as evidenced by the unexpected DI and panhypopituitarism. Due to the probability that asymptomatic pituitary metastases could become symptomatic after an invasive intervention, regular monitoring for signs of possible pituitary dysfunction is important.

Another factor that delayed diagnosis and treatment in this case was masked DI. Central DI results from deficient ADH synthesis or secretion, and its most common causes of central DI are benign or malignant neoplasms of the HPA [12]. Patients with central DI commonly present with polyuria and polydipsia. They may also suffer from severe dehydration and hypernatremia in cases of insufficient fluid intake. The symptoms and laboratory studies related to DI are masked by concomitant ACTH deficiency. Several reasons underlying masked DI have been proposed. Adrenal insufficiency impairs renal free water clearance [3]. It may also upregulate corticotropin-releasing hormone (CRH), which stimulates both ADH and ACTH [13]. Finally, since cortisol induces ADH resistance of the V_2_ receptor, adrenal insufficiency may amplify the ADH effects [14].

In this study, upon initiation of glucocorticoid replacement therapy, DI symptoms, such as polyuria, appeared. Therefore, the normal plasma sodium levels and daily urinary output observed until glucocorticoid replacement were due to masked DI. Glucocorticoid replacement is effective for the diagnosis of masked DI because glucocorticoid replacement increases the sensitivity for diagnosis in cases of pituitary metastasis [2]. The fatigue and lethargy experienced by the patient after the biopsy surgery might be related to panhypopituitarism because these symptoms are common complaints among patients with this condition [2]. In pituitary metastases, even in asymptomatic cases, physicians should not overlook these nonspecific symptoms. Prolonged fatigue and lethargy in cases of pituitary metastases could be a potential warning sign of masked DI and should be monitored to avoid misdiagnosis and delayed treatment.

Another case report of pituitary metastasis discussed severe hyponatremia, which was induced by secondary adrenal insufficiency accompanied by DI [3]. In this case, a decreasing trend in plasma sodium levels was observed. Our patient may have suffered from hyponatremia if glucocorticoid replacement therapy had not been administered.

Previous studies have reported that most pituitary metastases occur in the posterior lobe [1, 2]. This could be because direct perfusion from the inferior hypophyseal arteries into the posterior lobe might increase the likelihood of metastasis to the posterior lobe rather than the anterior lobe, which is indirectly perfused [2]. Moreover, this may account for the reports that DI is more common in pituitary metastases compared with other pituitary pathologies [15]. The key MRI findings related to DI are the absence of the normal T1 PPBS, as well as enhancement and thickening of the pituitary stalk [12]. In our case, we observed similar MRI findings before DI was detected. In retrospect, this should have been recognized as a sign of DI and should have prompted more thorough assessments and earlier treatment.

There are no standardized treatment guidelines for the management of pituitary metastases [5]. Although surgery for pituitary metastasis facilitates symptomatic relief for optic pathway compression, it does not impact survival [2, 7]. Conversely, survival was positively impacted by radiotherapy and chemotherapy [7]. A previous study demonstrated that there was no significant difference in estimated survival between radiation methods, such as conventional multiple fractionated radiation, Gamma Knife, and Cyberknife [16]. However, some studies have reported that stereotactic radiotherapy leads to improved survival compared with conventional radiotherapy [7], as well as improvements in DI and neurological symptoms [17, 18]. However, we chose to perform conventional multiple fractionated radiation because peritumoral edema of the right thalamus lesions progressed into the brainstem and the edema could have worsened after stereotactic radiotherapy [19]. The patient also underwent breast radiation therapy in combination with administration of oral molecularly targeted and cyclin-dependent kinases 4/6 inhibitor therapy.

The mean survival period after detecting pituitary metastasis is 10–14 months [1, 7]. In cases managed with conventional multiple fractionated radiation [7], such as in this case, the mean survival period is 10 months. Overall survival was significantly longer in recent studies than in studies before 2010 [7]. This may be due to advances in neuroimaging and oncological therapies, which facilitated more timely diagnoses and improved treatments. Therefore, for earlier diagnosis and efficient management in rare cases such as in this report, careful monitoring by a multidisciplinary team is required.

## Conclusions

In pituitary metastasis, it is important to consider that invasive examination or intervention may induce pituitary dysfunction, including DI or panhypopituitarism. Since ACTH insufficiency can mask DI, we must consider pituitary dysfunction even when urinary output and plasma sodium levels are normal. Careful monitoring of symptoms, including lethargy or fatigue, should be done to avoid delayed treatment.

## Data Availability

Data sharing is not applicable to this article as no datasets were generated or analyzed during the current study.

## Abbreviations

ACTH: Adrenocorticotropic hormone
ADH: Antidiuretic hormone
CRH: Corticotropin releasing hormone
DI: Diabetes insipidus
HPA: Hypothalamic–pituitary axis
MRI: Magnetic resonance imaging
PPBS: Posterior pituitary bright spot
T1WI: T1-weighted imaging
T2WI: T2-weighted imaging
